# Reclassification of the biocontrol agents *Bacillus subtilis* BY-2 and Tu-100 as *Bacillus velezensis* and insights into the genomic and specialized metabolite diversity of the species

**DOI:** 10.1099/mic.0.000986

**Published:** 2020-11-09

**Authors:** Alex J. Mullins, Yinshui Li, Lu Qin, Xiaojia Hu, Lihua Xie, Chiming Gu, Eshwar Mahenthiralingam, Xing Liao, Gordon Webster

**Affiliations:** ^1^​ Microbiomes, Microbes and Informatics Group, Organisms and Environment Division, School of Biosciences, Cardiff University, Cardiff, CF10 3AX, Wales, UK; ^2^​ Oil Crops Research Institute of Chinese Academy of Agricultural Sciences, Key Laboratory of Biology and Genetic Improvement of Oil Crops, Ministry of Agriculture and Rural Affairs, Wuhan 430062, PR China

**Keywords:** *Bacillus velezensis*, biocontrol, genomics, biosynthetic gene clusters

## Abstract

The genomes of two historical *Bacillus* species strains isolated from the roots of oilseed rape and used routinely in PR China as biocontrol agents to suppress *Sclerotinia* disease were sequenced. Average nucleotide identity (ANI) and digital DNA–DNA hybridization analyses demonstrated that they were originally misclassified as *Bacillus subtilis* and now belong to the bacterial species *Bacillus velezensis *. A broader ANI analysis of available *Bacillus* genomes identified 292 *B. velezensis* genomes that were then subjected to core gene analysis and phylogenomics. Prediction and dereplication of specialized metabolite biosynthetic gene clusters (BGCs) defined the prevalence of multiple antimicrobial-associated BGCs and highlighted the natural product potential of *B. velezensis *. By defining the core and accessory antimicrobial biosynthetic capacity of the species, we offer an in-depth understanding of *B. velezensis* natural product capacity to facilitate the selection and testing of *B. velezensis* strains for use as biological control agents.

## Introduction

Phytopathogens are a major constraint on global food production, causing plant disease in the field and during post-harvest storage. The over-reliance on synthetic pesticides in modern agriculture and the damage they cause to soil fertility, the accumulation of toxic residues and the emergence of pathogen resistance have led to a need for more sustainable disease management practices [1]. Biological control of plant pathogens using naturally occurring antagonistic micro-organisms (biopesticides) is one such practice, and it has recently attracted renewed research interest due to the requirement for environmentally friendly options [2]. Several micro-organisms have been developed as biopesticides and used routinely in the field, and among the most successful are members of the *Bacillus subtilis* species complex, known to suppress disease caused by bacterial, fungal, viral and nematode plant pathogens [2–5].

The important *B. subtilis* species complex [6] includes approximately 20 closely related species [7] and belongs to the phylum *Firmicutes *. However, the taxonomy is notoriously difficult to discriminate based on traditional phenotypic and 16S rRNA gene sequencing methods [7, 8]. For example, comparison of the complete 16S rRNA gene sequences of *Bacillus amyloliquefaciens* DSM 7^T^ and *B. subtilis* 168^T^ revealed >99 % sequence identity [7]. The taxonomy of the *B. subtilis* species complex was recently updated and clarified through genomic analysis [7, 9–11]. The complex now includes four monophyletic groups: the clade I *B. subtilis* group; the clade II *B. amyloliquefaciens* group; the clade III *Bacillus licheniformis* group; and the clade IV *Bacillus pumilus* group [7]. The clade II *B. amyloliquefaciens* group contains strains that are more proficient at rhizosphere colonization and biocontrol than other members of the *B. subtilis* species complex [8, 12], and average nucleotide identity (ANI), digital DNA–DNA hybridization (dDDH) and core gene phylogeny demonstrate that clade II comprises three discrete but closely related species: *B. amyloliquefaciens *, *Bacillus velezensis* and *Bacillus siamensis* [7, 13].

From the biotechnological point of view, a key feature of the *B. amyloliquefaciens* group is their diverse metabolism and the ability to produce a wide range of different antagonistic compounds resulting in broad antimicrobial activity [2, 13, 14]. The model biocontrol bacterium *B. velezensis* FZB42 (formerly *B. amyloliquefaciens* subspecies *plantarum *) has a large part of its genome dedicated to the synthesis of specialized metabolites, with the ability to produce numerous structurally diverse antimicrobial compounds, including cyclic lipopeptides (e.g. surfactin, bacillomycin-D, fengycin, bacillibactin), polyketides (e.g. macrolactin, bacillaene, difficidin), siderophores and bacteriocins [5, 15–17]. In comparison with *B. amyloliquefaciens* and *B. siamensis *, the species *B. velezensis* has a higher number of biosynthetic genes involved in specialized metabolite production [7, 13].

In this study we aimed to sequence the genomes of two important *Bacillus* species strains (BY-2 and Tu-100) used routinely in PR China as biocontrol agents for the pathogen of oilseed rape, *Sclerotinia sclerotiorum* [18], which causes a disease of major importance in PR China [19], with annual yield losses of 10–80 % [18]. Previously both strains had been identified as *B. subtilis* [20, 21], but via genomic analysis we have revised their taxonomic status within the *B. subtilis* species complex as *B. velezensis* positioned in the clade II *B. amyloliquefaciens* group. In addition, the two biocontrol strains were analysed for their specialized metabolite biosynthetic potential via genome mining alongside 290 additional *B. velezensis* genomes. Understanding the intricacies of the antimicrobial repertoire within *B. velezensis* will allow improved biocontrol formulations and applications to be developed.

## Methods

### Bacterial isolates

*Bacillus* strains BY-2 and Tu-100 were originally isolated from the internal root tissues [22] and the rhizosphere [23], respectively, of oilseed rape (*Brassica napus*) plants grown in field plots located in Wuhan, Hubei Province, PR China. Both strains were shown to control *Sclerotinia* disease on oilseed rape [4, 20, 21]. Strains were routinely maintained in lysogeny broth (LB) [24] supplemented with streptomycin (50 µg ml^−1^) and rifampicin (50 µg ml^−1^) for BY-2 and kanamycin (30 µg ml^−1^) and rifampicin (50 µg ml^−1^) for Tu-100 to prevent contamination; BY-2 and Tu-100 are naturally resistant to these antibiotics at these concentrations. Strains BY-2 and Tu-100 are held in the Agricultural Culture Collection of China (Beijing, PR China) as ACCC 06137 and ACCC 05846, respectively.

### Genome sequencing, assembly and annotation

Strains BY-2 and Tu-100 were grown in LB at 28 °C in 250 ml volume Erlenmeyer flasks and shaken at 150 r.p.m. for 48 h. Genomic DNA was then extracted using a modified cetyltrimethylammonium bromide (CTAB) extraction method as described previously [25], and DNA concentration, quality and integrity were determined with a Qubit Fluorometer (Invitrogen) and a NanoDrop Spectrophotometer (Thermo Scientific). Sequencing libraries were generated using the TruSeq DNA Sample Preparation kit (Illumina) and the Template Prep kit (Pacific Biosciences). Whole-genome data were then generated using an Illumina NovaSeq (paired-end, 2×150 bp) sequencing platform and a PacBio sequel system at Wuhan Yanxing Biotechnology Co., Ltd . PacBio reads were assembled into contigs by HGAP v4 implementing CANU v1.7.1. Contigs were then polished with Pilon v1.18 using the Illumina reads. Genes were predicted by GeneMarkS v4.32 [26] and classified using eggNOG v5.0 [27] according to the clusters of orthologous groups (COGs) functional classification. A genome map was drawn from coding sequences (CDSs) and non-coding RNA predictions by Wuhan Yanxing Biotechnology Co., Ltd using the CGView server [28].

### Genomic data source and analysis

All genomes submitted as *Bacillus* (Taxon ID 1386) were downloaded from the European Nucleotide Archive (ENA) using the ENA enaBrowserTools interface scripts (https://github.com/enasequence/enaBrowserTools). Analyses were performed using the Cloud Infrastructure for Microbial Bioinformatics (CLIMB) [29].

### Phylogenomics of *B. velezensis*

*B. velezensis* genomes were identified by comparing downloaded *Bacillus* genomes to the *B. velezensis* type strain NRRL B-41580^T^ using the average nucleotide identity analysis tool FastANI v1.2 [30]. Genomes with an ANI value ≥95 % were further verified using the alignment based (ANIm) ANI tool PyANI v0.2.9 [31] implementing a 95 % ANI species threshold [30, 32]. Genomes were annotated using Prokka v1.14.5, and the annotation files used to generate a core-gene alignment with Roary v3.13.0 [33] implementing MAFFT v7.455. A maximum-likelihood phylogeny was constructed using RAxML v8.2.12 with a GTR substitution matrix and GAMMA model of rate heterogeneity supported by 100 bootstraps. In addition, the Type (strain) Genome Server (TYGS) bioinformatics platform (https://tygs.dsmz.de) was used for whole genome-based taxonomic analysis of BY-2, Tu-100, FZB42 and KACC 13105 against type strains [34]. This platform provides both species assignment and digital DNA–DNA hybridization (dDDH) values to the closest type strain genomes available. The dDDH values (Table S1, available in the online version of this article) used in this study were calculated using formula *d*_4_ [sum of all identities found in high-scoring segment pairs (HSPs) divided by overall HSP length] [35]. The formula *d*_4_ is preferred as it is independent of genome length and robust against the use of incomplete draft genomes. Digital DNA-DNA hybridisation values of <70% were used to indicate different species [36].

### Biosynthetic gene cluster prediction and dereplication

Specialized metabolite biosynthetic gene clusters (BGCs) were predicted for *B. velezensis* genomes with antiSMASH 5.0.0. A k-mer based dereplication of BGCs was performed as described previously [37]. In brief, a pairwise comparison of BGC k-mers was achieved using the distance estimation tool Mash v2.2.2 with a variable distance threshold. Predicted BGCs with a sequence length below 6 kbp were excluded as these likely represented BGCs fragmented across multiple contigs. Due to the adjacency of the iturin BGC and fengycin BGC in *B. velezensis* genomes, a local blastn v2.9.0+ search was performed to confirm their distribution.

### *In vitro* fungal inhibition assays

*Bacillus* strains BY-2 and Tu-100 were grown in LB at 28 °C in 50 ml volume Erlenmeyer flasks and shaken at 150 r.p.m. After 48 h, growth bacteria were removed by centrifugation (5000 r.p.m. for 10 min; Eppendorf centrifuge 5427 R) and the spent media containing bacterial metabolites were sterilized using a 0.2 µm sterile filter. *In vitro* fungal inhibition assays were then conducted on 20 ml potato dextrose agar (PDA) contained in 9.0 cm plastic Petri dishes. Four holes were cut out of the PDA using a 5.0 mm diameter corer at equal distance around the outer part of the plate. To each assay plate (three replicate plates per fungi tested), the holes were filled with (i) 100 µl of iturin A (1.0 mg ml^−1^), (ii) 100 µl of BY-2 media, (iii) 100 µl of Tu-100 media and (iv) 100 µl of water as a control. A 5.0 mm diameter plug of leading-edge growth of *S. sclerotiorum* strain Ss-1 and *Rhizoctonia solani* strain Rs-2 was placed in the centre of the four treatments. Assay plates were incubated for a further 48 h at 22 °C (*S. sclerotiorum*) or 28 °C (*R. solani*), and the distance between the edge of the fungal colony and treatment was measured and averaged per plate.

## Results and discussion

### Reclassifying *B. subtilis* strains BY-2 and Tu-100 as *B. velezensis*

**Fig. 1.**
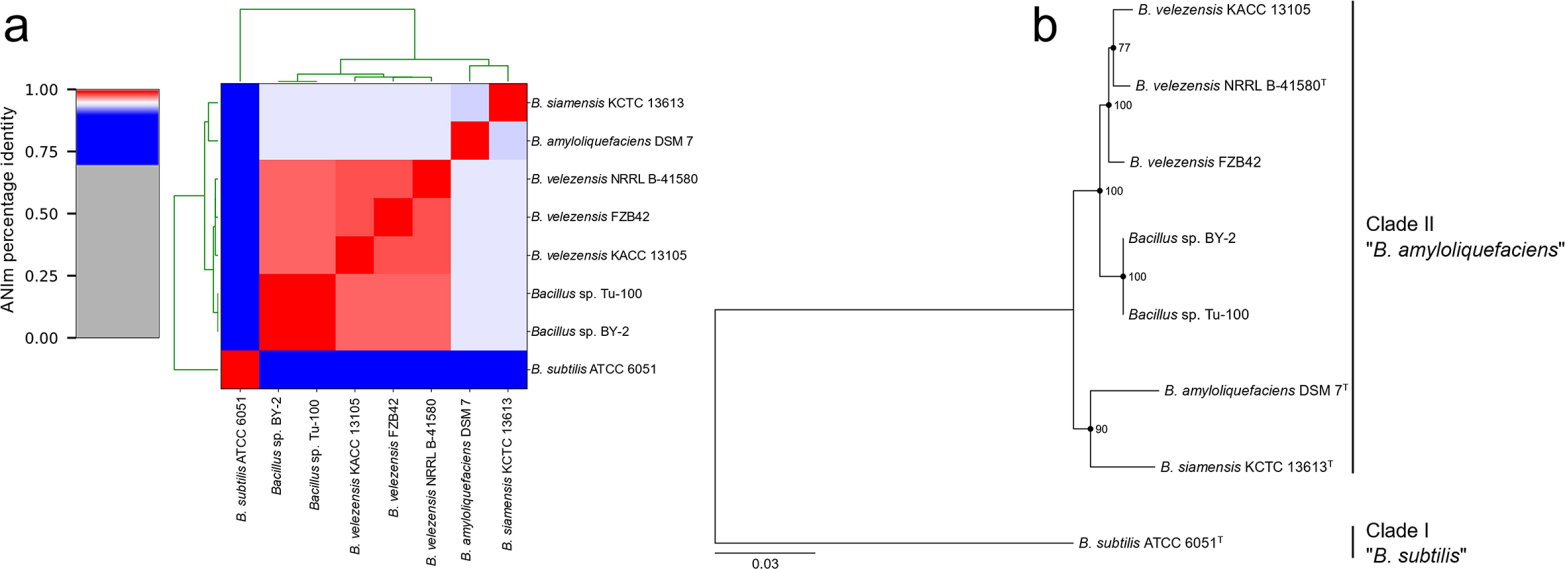
Pairwise average nucleotide identity analysis and core gene phylogeny. (a) *Bacillus* strains BY-2 and Tu-100 were compared to representatives of the *B. subtilis* species complex clade II *B. amyloliquefaciens* operational group and the *B. subtilis* type strain ATCC 6051^T^ (* B. subtilis* species complex clade I, operational group *B. subtilis *) using the ANI tool PyANI (ANIm). (b) A phylogeny was constructed using 313 core genes identified from the genomes used in the ANI analysis. Nodes are labelled with bootstrap values. Error bar represents substitutions per site.

The species classification of *Bacillus* strains BY-2 and Tu-100 was determined by comparing their genomes to more than 4800 *Bacillus* genomes using a k-mer ANI analysis. Both BY-2 and Tu-100 were similar (≥95 % ANI) to *Bacillus* genomes classified as *B. velezensis *. To confirm this classification, BY-2 and Tu-100 genomes were further compared by alignment-based ANI, digital DDH and core gene phylogenomics to type strains of the *B. amyloliquefaciens* clade [7] and *B. velezensis* strains formerly classified as different species (Fig. 1, Table S1). All three genome comparison methods gave congruent results and confirmed the placement of BY-2 and Tu-100 with *B. velezensis *, separate from other members of the *B. amyloliquefaciens* clade and the outgroup *B. subtilis* ATCC 6051^T^.

### The *B. velezensis* BY-2 and Tu-100 genomes

**Fig. 2.**
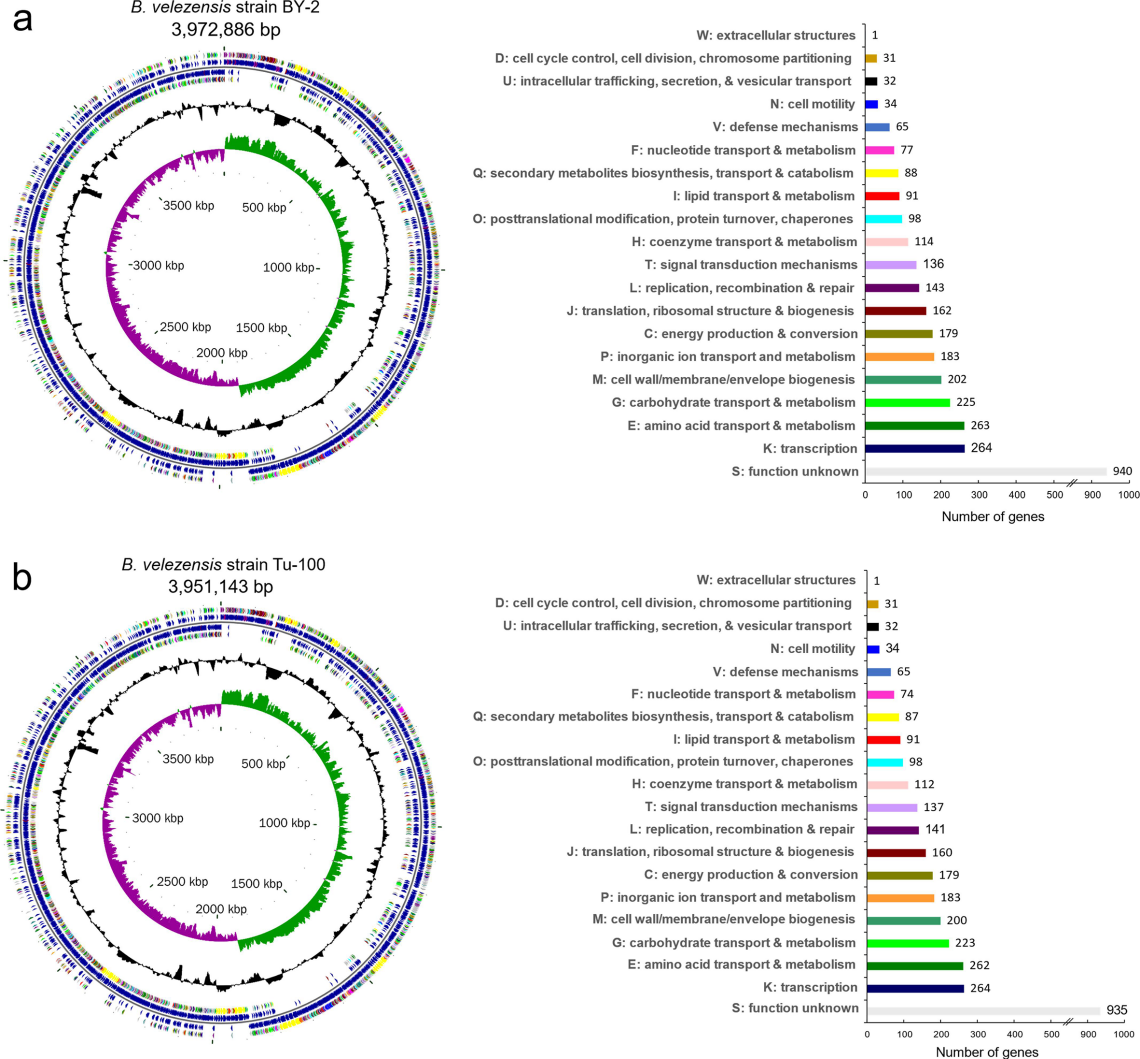
Visual representation and COG functional classification of the *B. velezensis* BY-2 and Tu-100 genomes. (a) *B. velezensis* BY-2 chromosome map and COG functional annotation of 3328 predicted proteins. (b) *B. velezensis* Tu-100 chromosome map and COG functional annotation of 3309 predicted proteins. From outer circle to the centre: CDSs on forward strand (coloured according to COG categories listed in the bar chart); all CDSs (blue) and RNA (brown/purple) genes on forward strand; all CDSs (blue) and RNA (brown/purple) genes on reverse strand; CDSs on reverse strand (coloured according to COG categories); GC content (black); GC skew (positive GC skew values are plotted in purple, and negative values are in green); scale bar. The map was generated using Circular Genome Viewer (CGView).

The genomes of *B. velezensis* strains BY-2 and Tu-100 consisted of a single circular chromosomal replicon that was 3.97 and 3.95 Mbp in size, respectively, with a G+C content of 46.5 % (Fig. 2, Table S2), and each also contained one 0.2 Mbp plasmid (Fig. S1). The genome metrics of both strains fitted within the size range of 3.81–4.24 Mbp and 45.9–46.8 % GC reported for other completed genomes of *B. velezensis* [38]. The BY-2 and Tu-100 genomes were predicted to carry 3915 and 3893 genes, respectively, of which approximately 85 % (3328 and 3309 CDSs, respectively) were classified as protein coding sequences with COG function (Table S3). Both BY-2 and Tu-100 carried 114 (2.9 %) and 108 (2.8 %) rRNA/tRNA coding genes, respectively, representing 9 and 8 copies each of the rRNA operon genes (5S, 16S and 23S rRNA genes; Fig. 2). The most abundant COG functions in the genomes of BY-2 and Tu-100 were associated with metabolism, and the proportion of genes related to metabolic functions was 36.7 % (1220/3328) and 36.6 % (1211/3309) of genes classified, respectively (Table S3). Specific functions of transcription (K), amino acid transport and metabolism (E), carbohydrate transport and metabolism (G), and cell wall/membrane/envelope biogenesis (M) were the most abundant COG categories in the genomes (Fig. 2, Table S3). Interestingly only 2.6 % of the genes (Fig. 2) in both genomes by COG were characterized as secondary metabolites biosynthesis, transport and catabolism (Q), whereas antiSMASH analysis predicted that 20.7 % of the genomes for both strains were involved in specialized metabolite production (Table S4). A similar discrepancy (2.8 and 20.2 %, respectively) in the two methods was also reported for the plant growth-promoting *B. velezensis* strain WRN014 [39]. This may be due to genes predicted within a BGC by antiSMASH not being successfully categorized or directly linked with secondary metabolism using the COG database. In addition, antiSMASH analysis of the model biocontrol strain *B. velezensis* FZB42 also suggests that it has 20.4 % of its genome dedicated to BGCs (data not shown) and this high percentage (approximately 1/5 of the genome) linked to specialized metabolism is a feature of *B. velezensis* strains [39].

### Genomic diversity of *B. velezensis* reveals sub-species structure

**Fig. 3.**
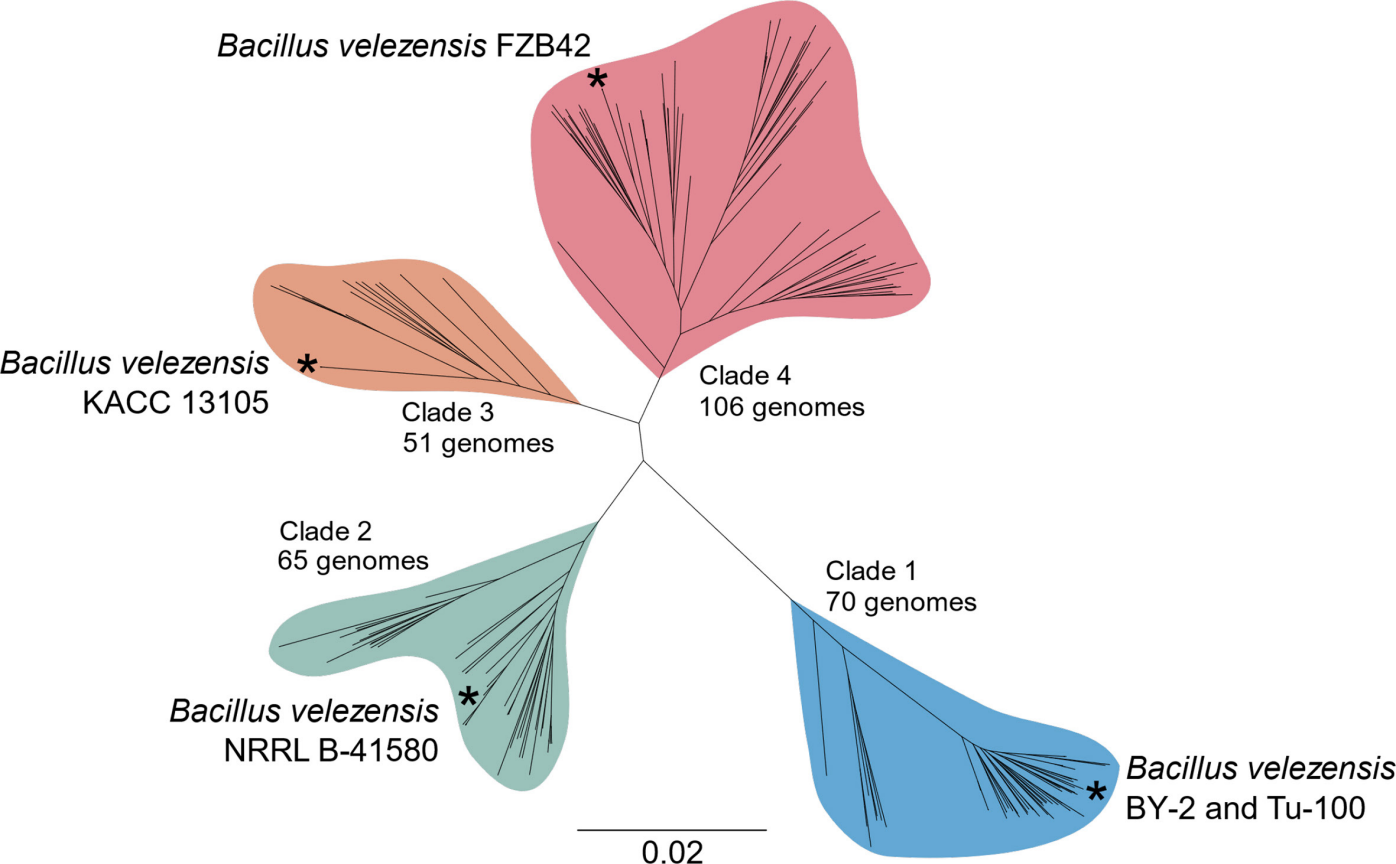
Unrooted core gene phylogeny of *B. velezensis *. The phylogenetic tree was constructed based on an alignment of 1301 core genes identified in 292 *B. velezensis* genomes. The phylogeny was divided into four broad clades and labelled: clade 4, *B. velezensis* FZB42 (formerly *B. amyloliquefaciens* subspecies *plantarum *); clade 3, *B. velezensis* KACC 13105 (formerly *B. methylotrophicus *); clade 2, *B. velezensis* type strain NRRL B-41580^T^; clade 1, *B. velezensis* strains BY-2 and Tu-100. The number of *B. velezensis* genomes in each clade is listed.

A core gene analysis and phylogeny were constructed to better understand the genomic diversity of the biological control species *B. velezensis *. An analysis of 292 genomes defined a total of 26 965 genes, and a core genome composed of 1301 genes. The average *B. velezensis* genome possessed 3871 genes, indicating that, on average, 66.4 % of the genes in each strain represent accessory functions. The core gene phylogeny of the 292 genomes divided broadly into four *B. velezensis* clades, with the *B. velezensis* type strain NRRL B-41580^T^ and heterotypic synonym type strains, *B. velezensis* (formerly *B. methylotrophicus *) KACC 13105 and *B. velezensis* (formerly *B. amyloliquefaciens* subspecies *plantarum *) FZB42, representing distinct clades (Figs 3 and S2). The fourth group (clade 1) was occupied by the closely related *B. velezensis* strains BY-2 and Tu-100 (Figs 3 and S2).

### Specialized metabolite potential of *B. velezensis* based on biosynthetic gene cluster diversity

**Fig. 4.**
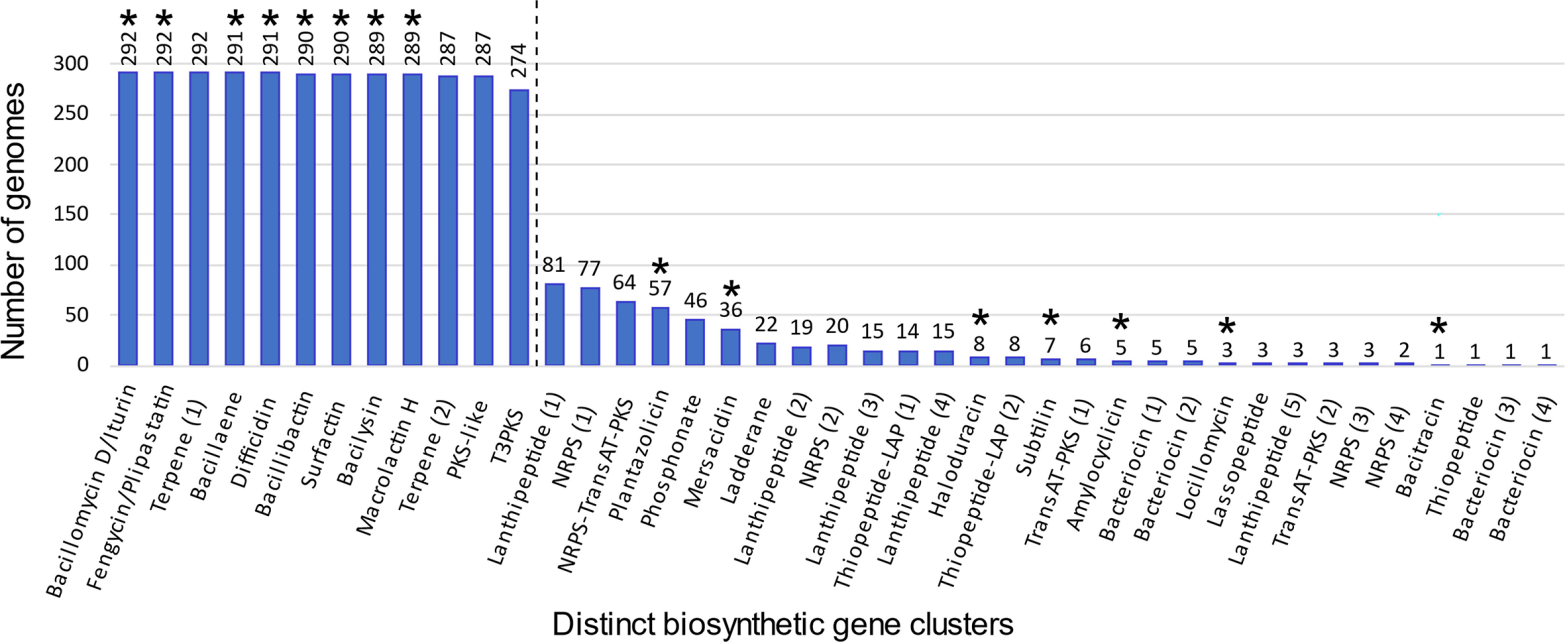
Occurrence of distinct biosynthetic gene clusters in *B. velezensis *. Following biosynthetic gene cluster (BGC) prediction and dereplication, a total of 41 distinct BGCs were identified across 292 *B. velezensis* genomes. NRPS, non-ribosomal peptide synthetase; PKS, polyketide synthase; LAP, linear azol(in)e-containing peptides. The presence of an asterisk indicates BGCs that have been characterized previously. The natural demarcation of core specialized metabolite BGCs occurring in >90 % strains is indicated by a dashed line.

Biosynthetic gene clusters were predicted for all 292 *B. velezensis* genomes to capture the potential of the species to synthesize specialized metabolites. Following the removal of fragmented BGCs, 4563 BGCs were defined and subsequently dereplicated into 41 distinct BGCs (Fig. 4; see File S1 for more details). Characterized specialized metabolite BGCs represented 15 of the 41 BGCs predicted across *B. velezensis *. A well-defined threshold was observed from this analysis that demarcated core BGCs, occurring in ≥90 % of *B. velezensis* strains, and accessory BGCs, occurring in <90 % of strains (Fig. 4). Only 12 BGCs (29 %) were defined as core, while the remaining 29 BGCs (71 %) constituted the accessory specialized metabolite capacity of the species. The majority of core BGCs (8 out of 12) were associated with known characterized antimicrobial metabolites, including iturin, fengycin, difficidin and macrolactin H. Other known antimicrobial BGCs were accessory and occurred less frequently in *B. velezensis* (Fig. 4). The plantazolicin and mersacidin loci were carried by 20 and 12 % of genomes, respectively; and the remaining five characterized BGCs (haloduracin, subtilin, amylocyclicin, locillomycin and bacitracin) each occurred in less than 3 % of genomes examined (Fig. 4). The most populous metabolite classes of uncharacterized BGCs were lanthipeptides, five BGCs; non-ribosomal peptide synthetases (NRPS), four BGCs; and bacteriocins, four BGCs. Interestingly, there was also evidence of two uncharacterized transAT-polyketide synthase (transAT-PKS)-encoding BGCs, and a hybrid NRPS-transAT-PKS-encoding BGC. A limited number of the accessory BGCs appeared to be *B. velezensis* clade-specific, with the majority exhibiting a broad distribution across the phylogeny (Fig. 3). The phosphonate BGC was restricted to *B. velezensis* clade 2 comprising the *B. velezensis* type strain NRRL B-41580^T^. However, each of the remaining clade-restricted metabolites, including the subtilin and locillomycin BGCs, only occurred in eight or fewer genomes and, as such, were sub-clade specific and not defining features of the broader phylogenetic clades. The two highly similar *B. velezensis* biocontrol strains BY-2 and Tu-100 possessed the same suite of specialized metabolite biosynthetic gene clusters as each other (Table S4). In addition to the 12 core BGCs defined above (Fig. 4), BY-2 and Tu-100 also carried 2 accessory BGCs, lanthipeptide (4) and the NRPS-transAT-PKS (Table S4). A similar study was conducted on the genus *Bacillus* analysing 1566 genomes, predicting 19 962 BGCs, and clustering similar BGCs [40]. The prevalence of characterized specialized metabolite BGCs, relative abundance of metabolite classes, and existence of uncharacterized BGCs across multiple species were highlighted to understand the natural product capacity of the genus [40]. This study focused mainly on the *Bacillus cereus* group species, which represented 90 % of the genomes analysed, and the remaining genomes outside the *B. cereus* group lacked representatives of *B. velezensis *.

### *B. velezensis* strains BY-2 and Tu-100 produce antifungal compounds

To confirm whether *B. velezensis* produces antifungal compounds *in vitro* an antagonism assay against two fungal plant pathogens was conducted. Clear bioactivity was observed for spent growth media of BY-2 and Tu-100 against the pathogens *S. sclerotiorum* and *R. solani* (Fig. S3). Interestingly, activity against the two pathogens was also observed for the pure lipopeptide iturin A (Fig. S3), a compound known to be produced by *B. velezensis* [14] and encoded for in both the BY-2 and Tu-100 genomes. Previously, it was shown by PCR amplification of the *ituC* and *ituD* that Tu-100 contained the iturin gene cluster, and it was possible to detect iturin in culture filtrates by using thin layer chromatography [20]. However, it is likely that the bioactivity of BY-2 and Tu-100 metabolites within the spent growth media was due to a mixture of compounds produced by several of the 14 BGCs detected in their genomes, which include known antifungal, antibacterial and nematocidal metabolites: iturin, fengycin, surfactin, bacillaene, difficidin and bacilysin (Fig. 4, Table S4) [41, 5].

### Summary

*B. velezensis* has emerged as a very promising biological control bacterium, with multiple studies highlighting its ability to control crop pathogens and produce a multitude of antimicrobial metabolites. This impressive specialized metabolite repertoire has been defined by the presence of the metabolites themselves or their corresponding biosynthetic gene clusters in specific strains [7, 5]. Other studies have investigated the broader distribution of metabolite classes, such as the iturinic lipopeptides, across the entire *B. subtilis* species complex [14], providing insights into the broader antimicrobial properties of *B. velezensis* and related species. In contrast, this study has explored the prevalence of known antimicrobial metabolite BGCs deposited in MiBIG across 292 *B. velezensis* genomes and highlighted the unexplored specialized metabolite potential through the prediction of uncharacterized BGCs. The detection of many known metabolites in *B. velezensis *, including iturin, plantazolicin and amylocyclicin, was congruent with the literature [5]. However, we also found evidence of other BGCs previously uncharacterized in *B. velezensis *, such as haloduracin and subtilin. Knowledge of the distribution of antimicrobial BGCs, and core versus accessory BGCs, will contribute to identifying optimal strains for use as biological control agents and allow improved biocontrol formulations and applications to be developed.

## Supplementary Data

Supplementary material 1

Supplementary material 2
