# Supplementary material for: Reclassification of the biocontrol agents Bacillus subtilis BY-2 and Tu-100 as Bacillus velezensis and insights into the genomic and specialized metabolite diversity of the species
Source: Microbiology (Reading). 2020 Nov 9;166(12):1121–8. doi: 10.1099/mic.0.000986 (PMC7819358; doi:10.1099/mic.0.000986)
Supplement: Supplementary material 1 [file mic-166-1121-s001.pdf]

Supplementary Information

Reclassification of the biocontrol agents *Bacillus subtilis* BY-2 and Tu-100 as *Bacillus velezensis* and insights into the genomic and specialised metabolite diversity of the species

Alex J. Mullins[2†], Yinshui Li[1†], Lu Qin[1], Xiaojia Hu[1], Lihua Xie[1], Chiming Gu[1], Eshwar Mahenthiralingam[2], Xing Liao[1*], Gordon Webster[2*]

†Equal contribution

[1]Oil Crops Research Institute of Chinese Academy of Agricultural Sciences, Key Laboratory of Biology and Genetic Improvement of Oil Crops, Ministry of Agriculture and Rural Affairs, Wuhan 430062, China.

[2]Microbiomes, Microbes and Informatics Group, Organisms and Environment Division, School of Biosciences, Cardiff University, Cardiff, Wales, CF10 3AX, UK.

∗Corresponding authors:

Gordon Webster, Microbiomes, Microbes and Informatics Group, Organisms and Environment Division, School of Biosciences, Cardiff University, Cardiff, Wales, CF10 3AX, UK.

Email: websterg@cardiff.ac.uk; Tel: +44 29 2087 5175.

Xing Liao, Oil Crops Research Institute of Chinese Academy of Agricultural Sciences, Key Laboratory of Biology and Genetic Improvement of Oil Crops, Ministry of Agriculture and Rural Affairs, Wuhan 430062, China. Email: liaox@oilcrops.cn; Tel: +86 27 86727093.

Supplementary Figure S1 Mullins, Li et al.

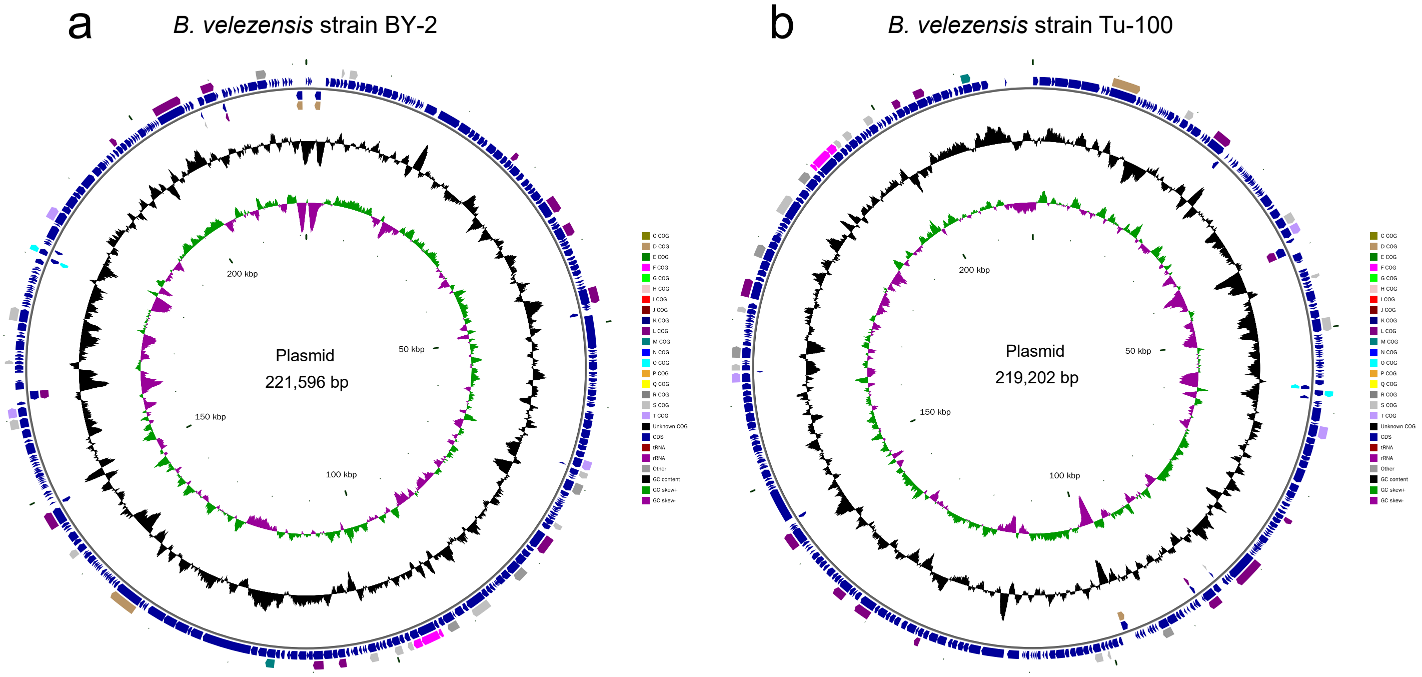

Supplementary Figure S1. Visual representation of *B. velezensis* (a) BY-2 and (b) Tu-100 plasmids. From outer circle to the centre: CDS on forward strand (coloured according to COG categories, see key); all CDS (blue) on forward strand; all CDS (blue) on reverse strand; CDS on reverse strand (coloured according to COG categories); GC content (black); GC skew (positive GC skew values are plotted in purple, and negative values are in green); scale bar. The map was generated using Circular Genome Viewer (CGView).

Supplementary Figure S2 Mullins, Li et al.

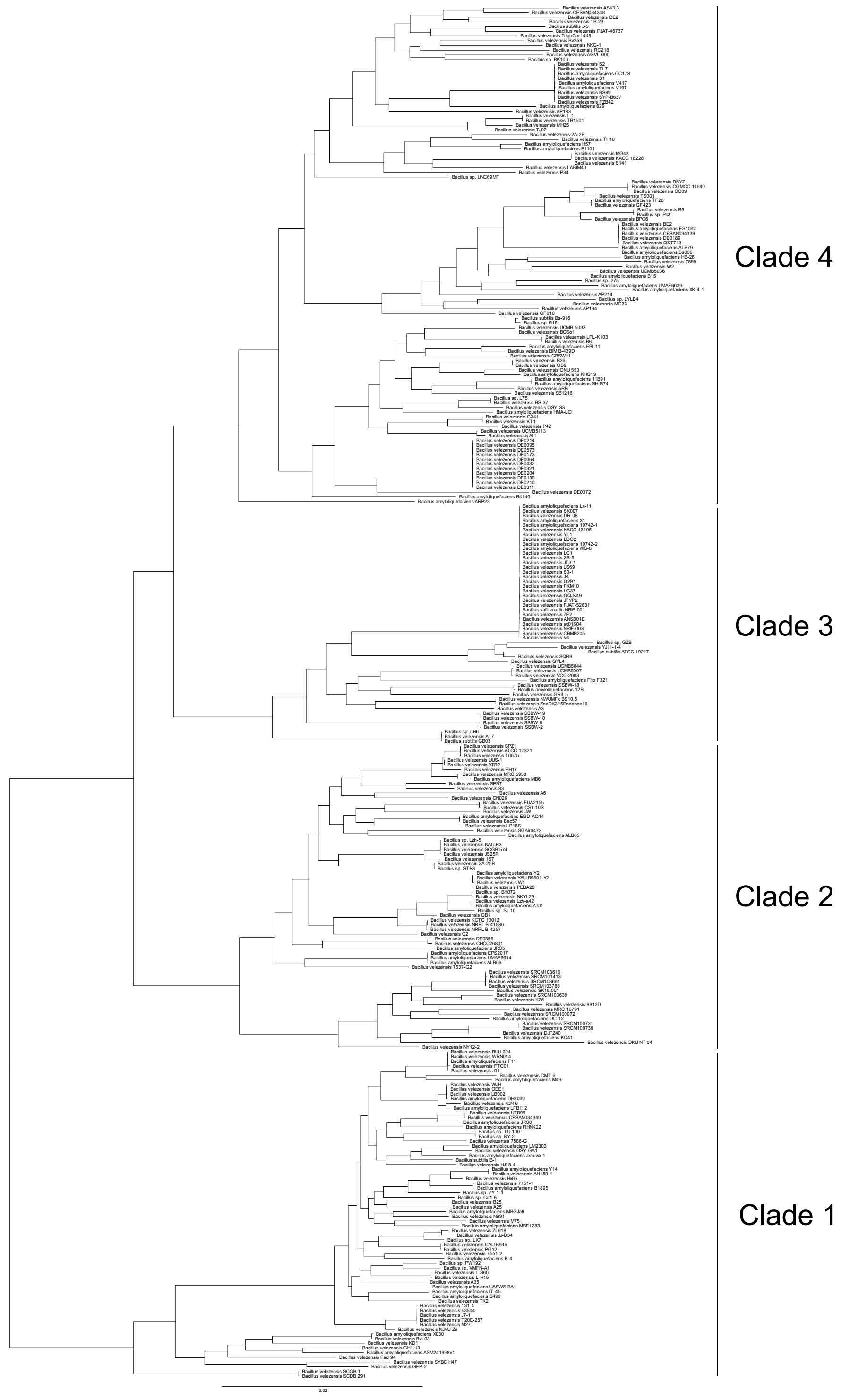

Supplementary Figure S2. Core gene phylogeny of *B. velezensis*. The phylogenetic tree was constructed based on an alignment of 1,301 core genes identified in 292 *B. velezensis* genomes. The rooting clade was determined by a secondary phylogeny including *Bacillus amyloliquefaciens* type strain DSM 7^{T} as an out group, based on an alignment of 1,267 core genes. The phylogeny was divided into four broad clades and labelled clades 1 to 4. Clade 1 contains *B. velezensis* strains BY-2 and Tu-100.

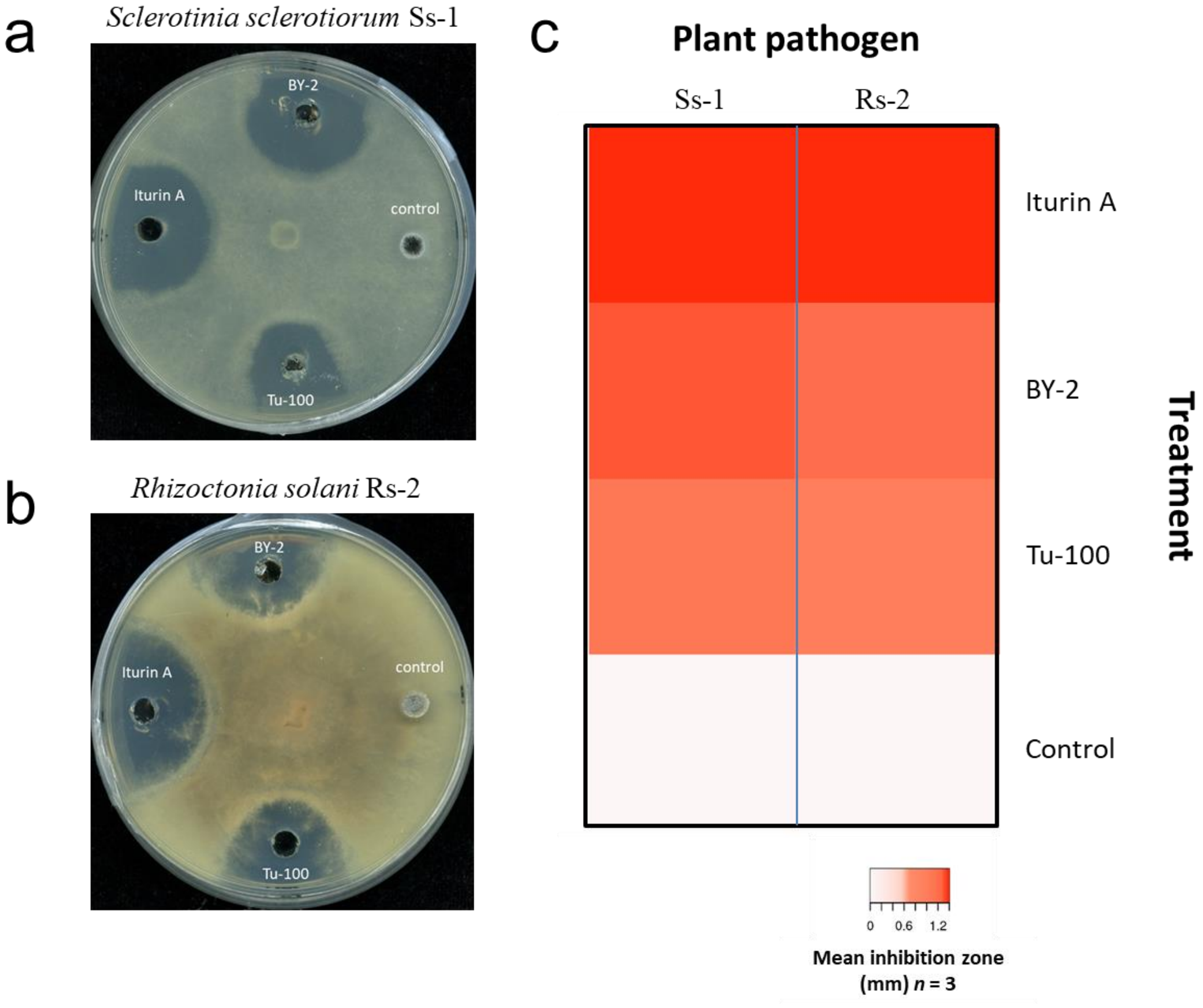

Supplementary Figure S3. Inhibition of plant fungal pathogens by *B. velezensis* strains BY-2 and Tu-100 in an *in vitro* antagonism assay. (a) Assay with *Sclerotinia sclerotiorum* Ss-1 and **(b)** assay with *Rhizoctonia solani* Rs-2. **(c)** Heat map showing the mean inhibition zone (mm) for replicate antagonism assays (n =3). Iturin A = 1.0 mg ml^{-1} iturin A (Sigma), control = sterile distilled water. BY-2 and Tu-100 = spent LB media from the respective 48 h-old culture of *B. velezensis* biocontrol strain.

Supplementary Table 1. Pairwise ANI and digital DDH between the two biocontrol strains and selected members of the *Bacillus subtilis* complex group, with their respective genome sizes.

**Genome (accession number)**	**Genome Size (Mbp)**	**Pairwise ANI (%)[a]**		**Pairwise dDDH (%)[b]**	
		**TU-100**	**BY-2**	**Tu-100**	**BY-2**
**Tu-100**	3.95	-	100	-	99.7
**BY-2**	3.97	100	-	99.7	-
***Bacillus velezensis* NRRL B-41580[T] (LLZC00000000)**	4.03	97.8	97.8	79.5	79.6
***Bacillus velezensis* FZB42 (CP000560)**	3.92	97.8	97.8	80.0	80.1
***Bacillus velezensis* KACC 13105 (JTKJ00000000)**	3.89	97.8	97.8	79.7	79.7
***Bacillus amyloliquefaciens* DSM 7[T] (FN597644)**	3.98	94.2	94.2	55.3	55.4
***Bacillus siamensis* KCTC 13613[T] (AJVF00000000)**	3.78	94.4	94.4	56.6	56.7
***Bacillus subtilis* ATCC 6051[T] (CP003329)**	4.29	84.4	84.4	20.8	20.8

[a] Average nucleotide identity (ANI) values <95% indicates different species (Richter & Rosselló-Móra, 2009; Jain *et al*., 2018).

[b] Digital DNA-DNA hybridisation (dDDH) values <70% indicates different species (Auch *et al*., 2010).

CONFIDENTIAL

Supplementary Table 2. Summary of genome assemblies for *Bacillus velezensis* biocontrol strains.

Genome	PacBio Sequencing depth (X)	Contig	Genome size (bp)	Sequence type	G + C content (%)	Total predicted CDS
**BY-2**	399	chromosome	3,972,886	circular	46.50	3915
		plasmid	221,596	circular	37.12	317
**Tu-100**	628	chromosome	3,951,143	circular	46.49	3893
		plasmid	219,202	circular	37.16	314

Prediction based on eggNOG (COG) database classification

CONFIDENTIAL

Supplementary Table 3. Summary of eggNOG (COG) classification of open reading frames (ORF) in the genome assemblies of *Bacillus velezensis* biocontrol strains.

COG categories	Category function	BY-2		Tu-100	
		ORF number	% classified ORF	ORF number	% classified ORF
**Cellular processing and signalling**					
D	Cell cycle control, cell division, chromosome partitioning	31	0.93	31	0.94
M	Cell wall/membrane/envelope biogenesis	202	6.07	200	6.04
N	Cell motility	34	1.02	34	1.03
O	Posttranslational modification, protein turnover, chaperones	98	2.94	98	2.96
T	Signal transduction mechanisms	136	4.09	137	4.14
U	Intracellular trafficking, secretion, and vesicular transport	32	0.96	32	0.97
V	Defense mechanisms	65	1.95	65	1.96
W	Extracellular structures	1	0.03	1	0.03
Y	Nuclear structure	0	0	0	0
Z	Cytoskeleton	0	0	0	0
-	Subtotal ORFs	599	18.00	598	18.07
**Information storage and processing**					
A	RNA processing and modification	0	0	0	0
B	Chromatin structure and dynamics	0	0	0	0
J	Translation, ribosomal structure, and biogenesis	162	4.87	160	4.84
K	Transcription	264	7.93	264	7.98
L	Replication, recombination, and repair	143	4.30	141	4.26
-	Subtotal ORFs	569	17.10	565	17.07
**Metabolism**					
C	Energy production and conversion	179	5.38	179	5.41
E	Amino acid transport and metabolism	263	7.90	262	7.92
F	Nucleotide transport and metabolism	77	2.31	74	2.24
G	Carbohydrate transport and metabolism	225	6.76	223	6.74
H	Coenzyme transport and metabolism	114	3.43	112	3.38
I	Lipid transport and metabolism	91	2.73	91	2.75
P	Inorganic ion transport and metabolism	183	5.50	183	5.53
Q	Secondary metabolites biosynthesis, transport, and catabolism	88	2.64	87	2.63
-	Subtotal ORFs	1,220	36.66	1,211	36.60
**Poorly characterised**					
R	General function prediction only	0	0	0	0
S	Function unknown	940	28.25	935	28.26
-	Subtotal ORFs	940	28.25	935	28.26
**Total**					
-	Total classified ORFS	3,328	100	3,309	100
-	Genes not in eggNOG database	587	-	584	-

CONFIDENTIAL

Supplementary Table 4. Biosynthetic gene clusters present in *B. velezensis* BY-2 and Tu-100.

[a]Predicted compound	Molecule type	Biosynthetic gene cluster	[b]Genome location in BY-2 (Kbp)	[b]Genome location in Tu-100 (Kbp)
Unknown	-	NRPS-transAT-PKS	196 - 273	174 - 252
**Surfactin**	lipopeptide	NRPS	345 - 410	324 - 388
**Unknown**	-	PKS-like	934 - 976	913 - 954
**Terpene (1)**	terpene	terpene	1,060 - 1,078	1,039 - 1,056
**Macrolactin H**	macrolide PK	transAT-PKS	1,422 - 1,510	1,400 - 1,488
**Bacillaene**	polyene PK	NRPS-transAT-PKS	1,731 - 1,841	1,709 - 1,819
**Fengycin/plipastatin**	lipopeptide	NRPS	1,897 - 1,966	1,875 - 1,945
**Iturin/bacillomycin D**	lipopeptide	transAT-PKS	1,966 - 2,031	1,945 - 2,009
**Terpene (2)**	terpene	terpene	2,095 - 2,117	2,073 - 2,095
**Unknown**	-	T3PKS	2,185 - 2,227	2,164 - 2,205
**Difficidin**	polyene PK	transAT-PKS	2,342 - 2,448	2,320 - 2,426
**Bacillibactin**	bacteriocin	NRPS	3,074 - 3,126	3,053 - 3,104
Lanthipeptide (4)	lanthipeptide	lanthipeptide	3,290 - 3,316	3,268 - 3,295
**Bacilysin**	dipeptide	other	3,638 - 3,679	3,616 - 3,658

[a] Names of compounds in bold are encoded by core genes of *B. velezensis* (see Figure 4).
[b] Genome locations are estimates based on antiSMASH output.
For BY-2 and Tu-100, approx. 821.3 Kbp = BGCs (20.7% of the genome).

CONFIDENTIAL
